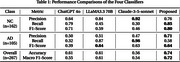# Dementia Prediction Using Hierarchical Attention and Evaluation of Context Quality

**DOI:** 10.1002/alz70857_102378

**Published:** 2025-12-25

**Authors:** Kyu‐haeng Lee, Seokbeom Lim, Ilju Lee, Ok Kim, Hyun Woo Jung, Sehwan Kim, Hee Jung Kim, Keunsoo Kang, Jung Jae Lee

**Affiliations:** ^1^ Dankook University, Yongin‐si, Gyeonggi‐do, Korea, Republic of (South); ^2^ Dannkook University, Yongin‐si, Gyeonggi‐do, Korea, Republic of (South); ^3^ Dankook University, Cheonan, Chungnam, Korea, Republic of (South)

## Abstract

**Background:**

A clinical interview is the first step in diagnosing dementia, as it helps determine whether the patient needs further evaluation. Large Language Models (LLMs) excel in understanding textual contexts and show significant promise in various domains, but their performance on real medical records has been less explored. The objective of this study is to evaluate the effectiveness of LLMs in predicting dementia solely from text‐based clinical notes.

**Method:**

We developed a deep learning‐based classifier for AD and Normal Control (NC) using clinical notes collected in South Korea (*n* = 1387, AD=542, NC=845), each categorized into 10 sections with up to 22 Question‐and‐Answer (QnA) pairs per category. To effectively extract the key information from the notes, we designed a hierarchical attention mechanism consisting of two layers: a Sentence‐Level Attention (SLA) layer and a Category‐Level Attention (CLA) layer. In the SLA layer, each QnA pair is contextualized using a pre‐trained DistilBERT model (while the proposed method remains compatible with any existing language model), with attention applied to combine responses within each category. The CLA layer then aggregates them while incorporating the “quality” of each category, determined by sentence specificity and topic relevance. The resulting final context vector is fed into a classification layer to predict an AD or NC label. The proposed model was trained on 80% of the dataset and evaluated on the remaining 20%. For comparison, we also evaluated the latest LLMs, including ChatGPT, LLaMA and Claude, as baseline classifiers, where the proposed method was not applied.

**Result:**

The proposed scheme outperforms the other approaches in most evaluations (Table 1). While the LLaMA and Claude models perform better on certain metrics, their overall results are inconsistent and lack reliability. In contrast, the proposed method delivers more robust and balanced outcomes, with an overall accuracy of 0.74 and an F1‐score of 0.72, both significantly higher than those achieved by the other three methods.

**Conclusion:**

Using a real‐world dataset, we demonstrate that analyzing clinical notes can serve as an effective method for early dementia screening. Furthermore, we observe that utilizing the quality of textual data enables the extraction of more nuanced contextual information, resulting in improved performance.